# Transcriptomic comparison of primary human lung cells with lung tissue samples and the human A549 lung cell line highlights cell type specific responses during infections with influenza A virus

**DOI:** 10.1038/s41598-022-24792-4

**Published:** 2022-11-29

**Authors:** Wilhelm Bertrams, Katja Hönzke, Benedikt Obermayer, Mario Tönnies, Torsten T. Bauer, Paul Schneider, Jens Neudecker, Jens C. Rückert, Thorsten Stiewe, Andrea Nist, Stephan Eggeling, Norbert Suttorp, Thorsten Wolff, Stefan Hippenstiel, Bernd Schmeck, Andreas C. Hocke

**Affiliations:** 1 Institute for Lung Research, Universities of Giessen and Marburg Lung Center, German Center for Lung Research (DZL), Philipps University Marburg, Hans-Meerwein-Strasse 2, 35043 Marburg, Germany; 2 Corporate Member of Freie Universität Berlin, Humboldt-Universität zu Berlin, and Berlin Institute of Health, Department of Internal Medicine/Infectious Diseases and Respiratory Medicine, Charité – Universitätsmedizin Berlin, Charitéplatz 1, 10117 ,Berlin, Germany; 3 Core Unit Bioinformatics, Berlin Institute of Health at Charité – Universitätsmedizin Berlin, Charitéplatz 1, 10117 Berlin, Germany; 4 HELIOS Clinic Emil von Behring, Department of Pneumology and Department of Thoracic Surgery, Chest Hospital Heckeshorn, Walterhöferstr. 11, 14165 Berlin, Germany; 5 Department of Thoracic Surgery, DRK Clinics, Drontheimer Strasse 39-40, 13359 Berlin, Germany; 6 Department of General, Visceral, Vascular and Thoracic Surgery, Universitätsmedizin Berlin, Charité Campus Mitte, 10117 Berlin, Germany; 7 Institute of Molecular Oncology, Genomics Core Facility, Member of the German Center for Lung Research (DZL), Philipps University, Marburg, Germany; 8 Department of Thoracic Surgery, Vivantes Clinics Neukölln, Rudower Straße 48 12351, Berlin, Germany; 9 Unit 17 “Influenza and Other Respiratory Viruses”, Robert Koch Institut, Seestrase 10, 13353 Berlin, Germany; 10 Department of Medicine, Pulmonary and Critical Care Medicine, University Medical Center Giessen and Marburg, Member of the German Center for Lung Research (DZL), Philipps-University, 35043 Marburg, Germany; 11 Center for Synthetic Microbiology (SYNMIKRO), Philipps-University, 35043 Marburg, Germany; 12 German Center for Infection Research (DZIF), Partner Site Giessen-Marburg-Langen, 35043 Marburg, Germany; 13 Institute for Lung Health (ILH), Giessen, Germany; 14 Core Facility - Extracellular Vesicles, Philipps-University Marburg, Marburg, Germany

**Keywords:** Immunology, Molecular biology

## Abstract

Influenza A virus (IAV) causes pandemics and annual epidemics of severe respiratory infections. A better understanding of the molecular regulation in tissue and cells upon IAV infection is needed to thoroughly understand pathogenesis. We analyzed IAV replication and gene expression induced by IAV strain H3N2 Panama in isolated primary human alveolar epithelial type II cells (AECIIs), the permanent A549 adenocarcinoma cell line, alveolar macrophages (AMs) and explanted human lung tissue by bulk RNA sequencing. Primary AECII exhibit in comparison to AM a broad set of strongly induced genes related to RIG-I and interferon (IFN) signaling. The response of AECII was partly mirrored in A549 cells. In human lung tissue, we observed induction of genes unlike in isolated cells. Viral RNA was used to correlate host cell gene expression changes with viral burden. While relative induction of key genes was similar, gene abundance was highest in AECII cells and AM, while weaker in the human lung (due to less IAV replication) and A549 cells (pointing to their limited suitability as a model). Correlation of host gene induction with viral burden allows a better understanding of the cell-type specific induction of pathways and a possible role of cellular crosstalk requiring intact tissue.

## Introduction

Pneumonia is the most severe consequence of respiratory infection. It remains one of the diseases with the highest mortality rates in children under five and older adults worldwide^1^. There has been little improvement in morbidity and mortality rates, despite vaccination strategies. One of the most common pneumonia-causing pathogens is influenza A virus (IAV)^2^. The rapid emergence of new IAV strains results in annual epidemics and occasional pandemics of acute respiratory illness in the human population^3^. For this reason, there is an urgent need to gain more knowledge about the molecular basis of the initial molecular host response, the cellular crosstalk and the pathogen-host interaction, particularly in human lung tissue, to understand most critical signaling pathways of this zoonotic disease.

The main target cells for IAV infection and replication in the human alveolus are alveolar type II epithelial cells (AECII)^4,5^. However, also alveolar macrophages (AM) are permissive, although the efficiency of virus replication is dependent on the viral strain and macrophage type^6^. Together, these cells play a critical role for the host defense and innate immune response by a coordinated cellular cross-talk^4,7^.

Studies in mice exhibit that AECII, compared to AM, activate a broader range of physiological responses including antiviral pathways in response to IAV infection^8^. However, differences between mice and men regarding cell differentiation and substantial diversification of lung cell types during mammalian evolution^9–11^ underline the importance of using human models that reflect both the tissue physiology and the clinical situation of the patient in the best possible way. Thus, we established a comprehensive map of differential gene expression in primary human AECIIs, AMs, and lung tissue explants. Previous studies have been done with various techniques and they were conducted at different stages of “omics”-development^12–14^, which makes comparison difficult. Also, they have not been done in parallel, nor with the same virus strain. In contrast, our study is the first systematic comparison between responses towards IAV infection for primary in vivo targets and the A549 cell line. Our analyses identified a shared core set of genes induced upon infection, including members of the *IFITM* and *OAS* gene family, but also highlighting cell-type specific differences with regard to relative induction upon infection, hence pathway enrichment, and absolute transcript abundance. The model of primary human lung tissue infected ex vivo provides further insight into the interplay between AMs and AECIIs and potential involvement of other cell types such as dendritic cells. AECII exhibited a broad set of strongly-induced genes, prominently related to RIG-I and interferon (IFN) signaling, which were induced to a lesser extent in the A549 cell line, which is widely used as IAV infection model. Although this adenocarcinoma-derived cell line expresses some characteristic features of ATII cells including synthesis of phospholipids, cytoplasmic lamellar bodies, and apical microvilli^15^ differences were shown in their response to virus infection including interferon and cell death signaling^16^. In conclusion, our study systematically defines the transcriptomic responses of the different sample types and allows direct comparison due to our stringent approach of parallel infection with the same virus strain.

## Materials and methods

### Cell culture and human lung tissue

A549 cells (ATCC, CCL-185) were cultured in Ham’s F12 medium and MDCKII (Dog, kidney) cells in Minimum Essential Medium supplemented with 10% fetal bovine serum and 2 mM l-glutamine at 37 °C in a 5% CO_2_ atmosphere. Cells were cultured up to passage 10 and then relaunched from frozen stock.

Peripheral human lung tissue was obtained from patients suffering from bronchial carcinoma. The study was approved by the ethics committee of the Charité – Universitätsmedizin Berlin (Project EA2/079/13). Written informed consent was obtained from all patients. Tumor-free peripheral lung tissue was processed as previously described^4,5,17^ and patient data are summarized in Table 1.

**Table 1:** Clinical patient data for used human lung tissue as far as known.

Sex	Age	Current smoking	Current diagnosis	Assay
**Lung tissue**				
na	na	na	na	RNA/Protein analysis
f	64	Yes	BC	RNA/Protein analysis
f	68	Yes	NSCLC	RNA/Protein analysis
f	66	Yes	NSCLC	Virus replication
m	45	No	BC	Virus replication
m	68	Yes	Squamous cell carcinoma	Virus replication
**AECII**				
na	na	na	na	RNA/Protein analysis
m	56	Yes	NSCLC	RNA/Protein analysis
na	na	na	na	RNA/Protein analysis
m	80	Yes	BC	Virus replication
f	73	Yes	AC	Virus replication
f	68	No	Cerebrovascular stroke	Virus replication
**AM**				
na	na	na	na	RNA/Protein analysis
m	64	Yes	BC	RNA/Protein analysis
f	68	No	Cerebrovascular stroke	RNA/Protein analysis
m	80	Yes	BC	Virus replication
f	73	Yes	AC	Virus replication
f	68	No	Cerebrovascular stroke	Virus replication

Lung tissue used for infection was free of tumor and taken from unaffected areas which were histologically considered as “normal”.

*BC* bronchial carcinoma, *AC* Adeno carcinoma, *NSCLC* non-small cell lung cancer, *na* data not available.

AMs were isolated by repeated perfusion of the human lung tissue with Hanks' balanced salt solution (HBSS). The resulting cells were seeded and cultured in Roswell Park Memorial Institute (RPMI) 1640 medium supplemented with 2% FCS and 2 mM l-glutamine under the conditions described above.

AECIIs were isolated as previously described^18^ with modifications. Briefly, human lung tissue was perfused to remove AMs as stated above and the remaining tissue was finely minced and digested with type I trypsin and DNase. The digested tissue was filtered and cells were incubated in hybridoma serum-free medium (SFM) to promote the differential adhesion of residual AMs. The cell fraction was then collected and layered on a Pancoll discontinuous gradient. Cells at the interfacial layer were collected, washed with HBSS and finally cells were seeded and cultured in RPMI 1649 medium supplemented with 10% FCS and 2 mM l-glutamine under the conditions described above^4^.

### Viral strain

The human seasonal IAV strain A/Panama/2007/1999 (Pan/99[H3N2]) was propagated in Madin-Darby canine kidney (MDCK) cells as previously described^5^. Virus stocks were titrated on MDCKII cells using a standard plaque assay.

### Ex vivo infection of human lung tissue and cells

Lung organ cultures were either mock-infected with infection medium (RPMI 1649 medium supplemented with 0.3% bovine serum albumin (BSA) and 2 mM l-glutamine) or inoculated with 1 × 10^6^ plaque-forming units (PFU) of Pan/99[H3N2] diluted in infection medium using a disposable syringe and needle as described^4,5,19^. Infection was done in a 200 µl volume of infection or control medium per 100 mg lung tissue. After 1.5 h, excess virus was removed by 3 washing steps with PBS. The lung tissue explants were incubated for 24 h at 37 °C in a 5% CO_2_ atmosphere. A549 cells, AMs and AECIIs were either mock-infected with infection medium or challenged with Pan/99[H3N2] at a multiplicity of infection (MOI) of 1. After 1.5 h, excess virus was removed by 3 washing steps with PBS. Cells were incubated for 24 h at 37 °C in a 5% CO_2_ atmosphere.

### Infectious particle quantification

Infectious particles were quantified by plaque titration on MDCKII cells. Briefly, cell monolayers were seeded in 24-well plates, incubated with virus-containing cell culture supernatants and overlaid with 1.2% Avicel in appropriate medium. After 48 h cells were washed two times with PBS and plaques were fixed and visualized by staining with crystal violet.

### Isolation of total RNA and cDNA synthesis

Total RNA was isolated using the Direct-zol RNA Kit (Zymo Research, Germany) according to the manufacturer’s instructions. Human lung tissue was transferred to Lysing Matrix D tubes (MP Biomedicals, Germany) in 500 μl TRIzol reagent. The tissue was lysed using a FastPrep-24 device (MP Biomedicals) by applying four rounds of tissue lysis at default settings (4 m/s, 20 s). Cells were directly lysed in 500 μl TRIzol reagent. The homogenates were centrifuged and 0.5 µg of RNA purified from the supernatant was reverse transcribed for the profiling of IFN subtypes using the RT^2^ Profiler Kit (Qiagen, Germany).

### RNA sequencing

RNA from infected samples was purified as described above and 500 ng was used for library preparation and sequencing on an Illumina HiSeq 1500 in the Philipps University Marburg Sequencing Core Facility. Reads were mapped using CLC Workbench v10.0 (Qiagen). Viral reads were detected by mapping against a hybrid virus/human reference. Differential gene expression was computed as logarithmic fold change with the DeSeq2 Package^20^ in *R* v3.5.2. Genes were considered differentially expressed at *p*_adj._ < 0.05 (Benjamini–Hochberg corrected for multiple testing)_._ For the representation of gene abundance as an important additional information to interpret fold-change data, transcripts per million (TPM) values were computed, z-score transformed and represented in heatmaps. Pathway enrichment analysis was carried out using the *R* package gprofiler2^21^ and Qiagen Ingenuity Pathway Analysis. Transcription factor enrichment was calculated using CiiiDER^22^. Correlation analysis was carried out by computing the Pearson correlation coefficient of genes with a selected single gene or composite ratio of viral NA and host ACTB. Data are deposited at NCBI GEO under the accession number GSE206606. All gene groups utilized are listed in table S1.

### ELISA

Cell-free and tissue-free supernatants were assayed 24 h following infection for release of Interferon α, β, γ, and λ1 by ELISA (PBL Assay Science, USA; eBioscience, USA) following the manufacturer´s instructions.

### Statistics

Statistical analysis not pertaining to RNA sequencing was carried out using PRISM v6 (GraphPad Software, USA) and data are presented as the mean ± standard error (SEM) of at least three donors representing independent experiments.

## Results

### Transcriptional analysis reveals that IAV infection induces numerous pathways exclusively in primary AECIIs compared to A549 cells

**Figure 1:**
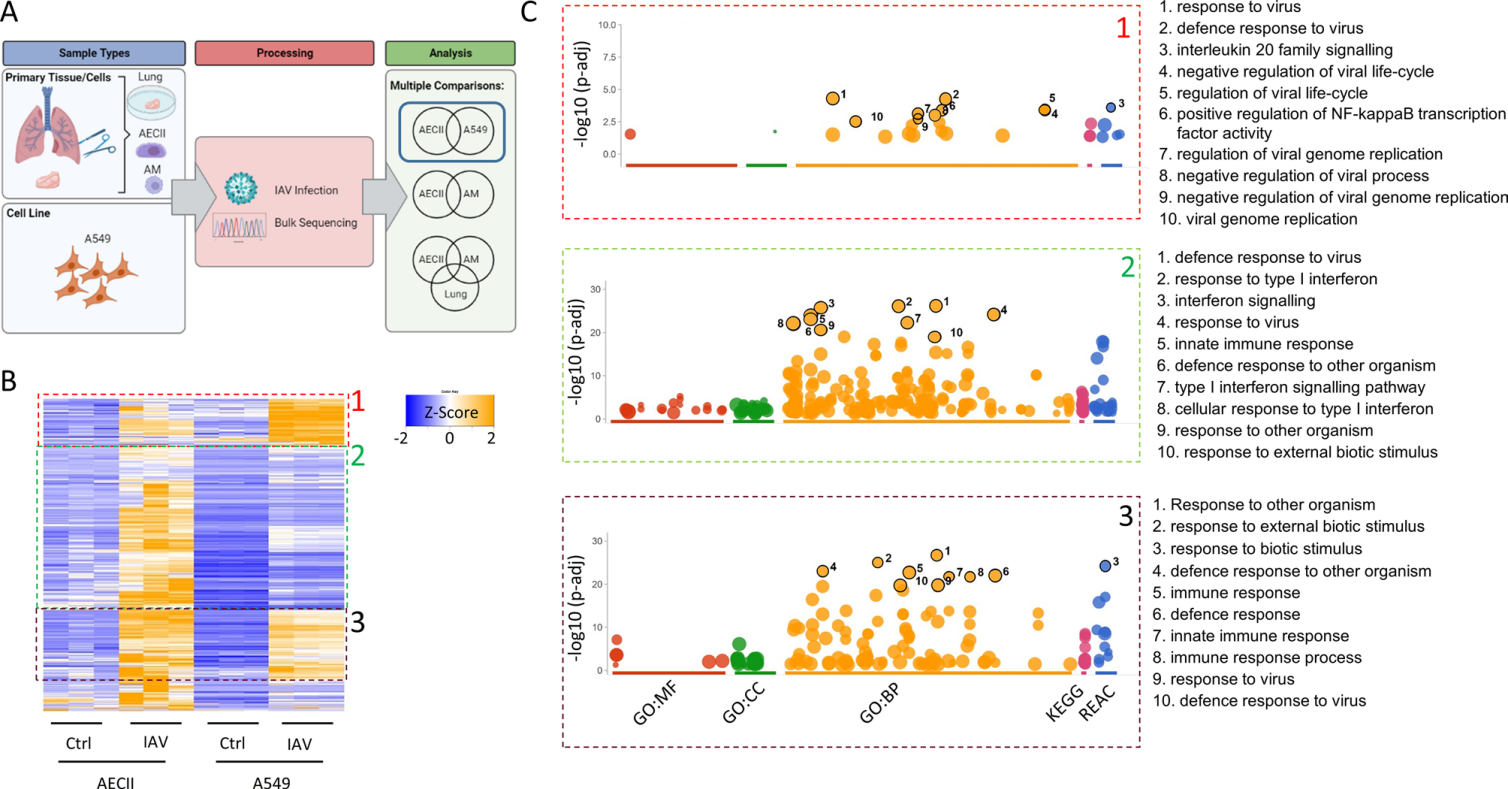
Comparative transcriptional analysis of AECIIs and A549 cells during IAV infection. (**A**) 592 genes are exclusively induced in AECII cells, 503 genes are exclusively induced in A549 cells, and there is a shared set of 468 induced genes. (**B**) The corresponding mRNAs of the shared set differ in abundance and relative induction between the two cell types (1 = similar genes, 2 = more pronounced induction in AECIIs, 3 = more pronounced induction in A549 cells). (**C**) Gene Ontology (GO) categories (BP = biological process, CC = cell compartment, MP = molecular process) and database classifications (KEGG = Kyoto Encyclopedia of Genes and Genomes, REAC = reactome database) highlights different pathway enrichment patterns for these three gene sets. Parts of this Figure were made with Biorender.

To validate the permissiveness of all four culture models for IAV we infected human lung tissue explants, AECII cells, AM and A549 cells with the seasonal IAV strain Pan/99[H3N2] and observed efficient replication of the virus in all used host systems (Fig. S1). We then investigated the cell-specific responses to IAV infection by initially comparing virus-modulated genes in A549 cells and AECIIs. We identified 592 genes that were exclusively upregulated in AECIIs, 503 genes that were exclusively upregulated in A549 cells, and 468 genes that were upregulated in both (Fig. 1A). Among the 468 shared genes, z-score hierarchical clustering revealed that many of the corresponding mRNAs were less abundant in A549 cells than in AECIIs, under resting conditions and during infection (Fig. 1B). A smaller number of mRNAs were similarly abundant in both cell types (fraction 3) or more abundant in A549 cells (fraction 1). Pathway enrichment analysis yielded very similar results (Fig. 1C, Figure S2). We next investigated pathway enrichment signatures in genes that were exclusively upregulated in one particular cell type, revealing pathways that were only activated in AECIIs (Fig. S3A). Closer investigation of two representative pathways (*antiviral mechanism by IFN-stimulated genes* and *RIG-I like receptor signaling*) indicated that only a part of the genes were induced by IAV infection in A549 cells. The Type I and II IFN response of A549 cells consequently appears to be diminished relative to AECIIs shown in an independent experiment on the RNA (Fig. S3B) and partly on the protein level (Fig. S7B). Of note, among the genes which are more abundant in A549 is *e.g. TRIM31*, which has been described as oncogenic ^23^.

### Induction of common antiviral responses in AMs and AECIIs as well as AECII-specific pathways

**Figure 2:**
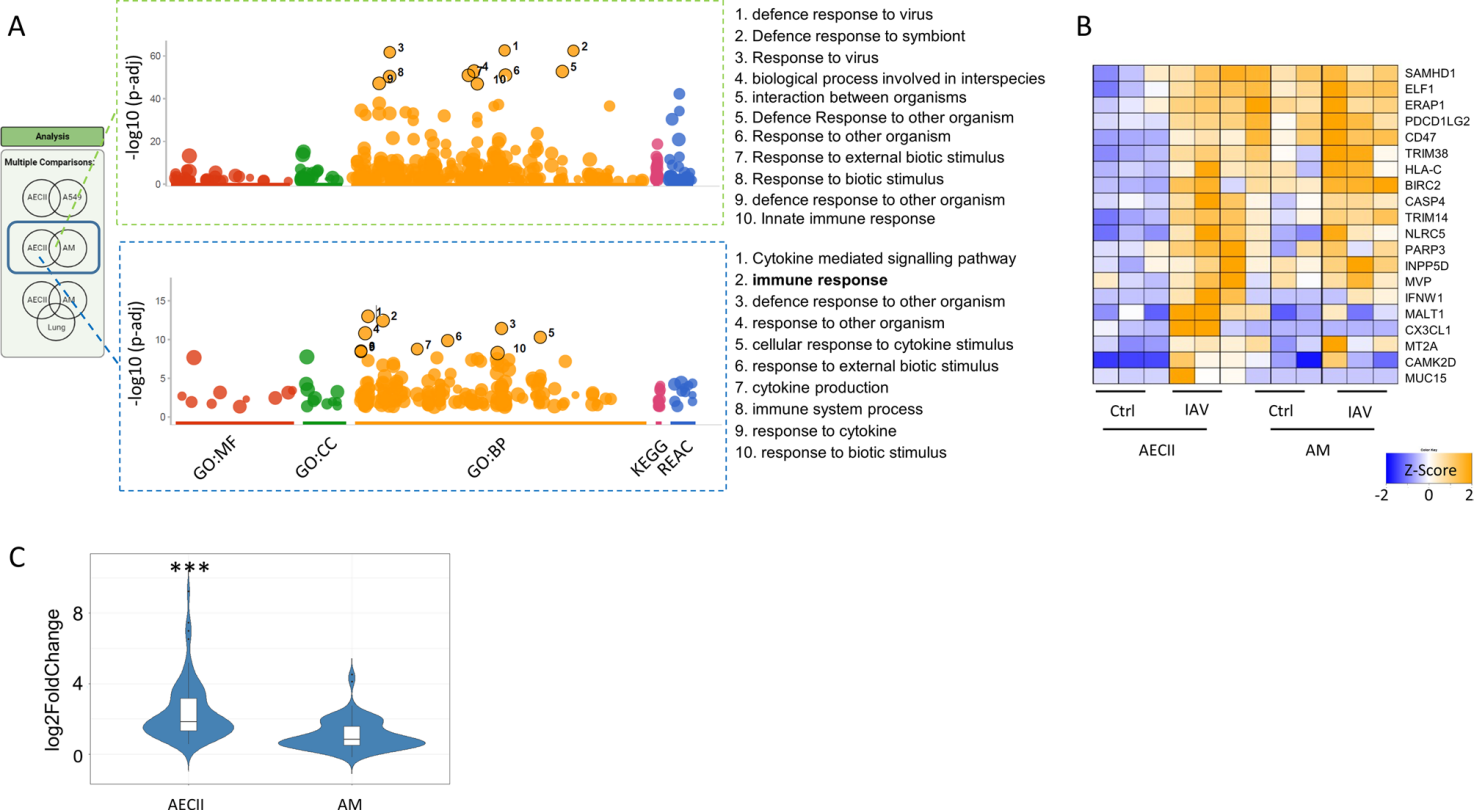
Comparison of virus-induced genes between AM and AEC yielded a strong shared antiviral response and a set of enriched pathways that was driven by AEC-specific genes (**A**). The top 10 of these pathways as ranked by p value are shown. Exemplary candidates from the GO term “Immune Response” show that the lack of virus induced genes from this pathway in AM is due to a high basal abundance of these genes. Z- scores were calculated on the basis of transcripts per million (TPM) (**B**). Broad lack of relative gene induction in AM was confirmed when investigating all 79 genes from this pathway that were part of the AECII-exclusive Venn section (**C**). Statistical significance was assessed by ANOVA with tukey correction for multiple testing. ***p < 0.001. Classifications: BP = biological process, CC = cell compartment, MP = molecular process, KEGG = Kyoto Encyclopedia of Genes and Genomes, REAC = reactome database. Parts of this Figure were made with Biorender.

Next, we characterized the cell-specific responses to IAV strain Pan/99[H3N2] in more detail by comparing AMs and AECIIs. As expected, the shared upregulated genes (n = 620) revealed a strong enrichment of defense responses and IFN pathways. Many more genes were upregulated specifically in AMs (n = 1061) than in AECIIs (n = 440) (Fig. 2A, Fig. S4). Analysis of the AM-specific genes yielded only weak pathway enrichment (not shown), whereas the AECII-specific genes showed a clear enrichment of the Gene Ontology (GO) terms *cytokine-mediated signaling pathway* and *immune response*, among others. A set of 79 genes (Table S1) associated with the GO term *immune response* was found in the AECII-exclusive Venn diagram section from panel 2A, which were more abundant in AM (Fig. 2B). Their mean relative fold change upon infection was significantly weaker in AM vs. AECII (Fig. 2C).

### IAV triggers a broad transcriptional response in primary human cells and whole lung tissue, which can be further resolved by mRNA abundance

**Figure 3:**
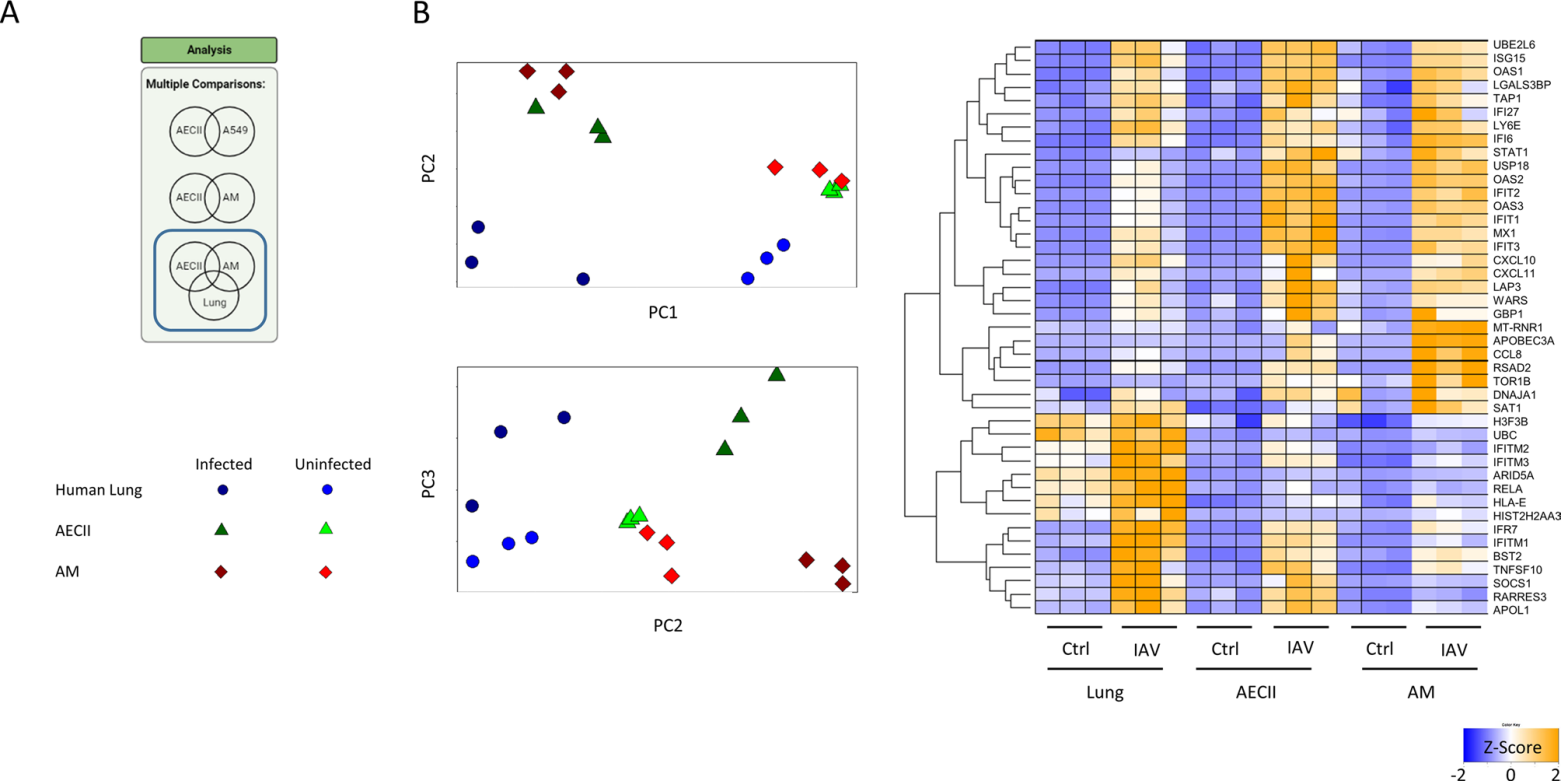
Infection with IAV leads to a broad transcriptional response in primary human cells and in whole lung tissue. (**A**) A set of 302 induced genes is shared as a common response between all cell types (a core set of genes), but the expression level of these genes differs across cell types. Principal component analysis (PCA) was performed to resolve the contribution of individual genes from this core signature to the different transcriptional landscapes. PC1 separated control samples from infected samples, PC2 separated AECII from human lung samples, and PC3 separated AECII from AM. Z-scores of genes driving sample separation were calculated on the basis of transcripts per million (TPM) and are shown as a heatmap (**B**). Parts of this Figure were made with Biorender.

We next analyzed primary human lung samples to reveal crosstalk-dependent gene expression between AECII and AM. While the analysis of cell-exclusive genes yielded pathway enrichment patterns with low-confidence (Figure S5), we identified 302 genes that were upregulated in the human lung, AECIIs and AMs (Fig. 3A). Principal component analysis (PCA) of these genes revealed good separation of the different sample types. Principal component 1 (PC1) clustered samples according to infection status, PC2 separated human lung tissue from AECII, and PC3 separated AECII from AM. The union of the 30 genes with the highest loading per principal component are illustrated as heatmap, showing abundance differences in the respective sample types. *IFITM1*, *IFITM2* and *IFITM3*, e.g., were genes with high abundance in the human lung. Conversely, *OAS1*, *OAS2* and *OAS3*, e.g., showed a reduced abundance in the human lung compared to AECII and AM. (Fig. 3B).

### AECII secrete a broader range of IFNs than AMs and their IFN target genes are induced more strongly by viral infection

**Figure 4:**
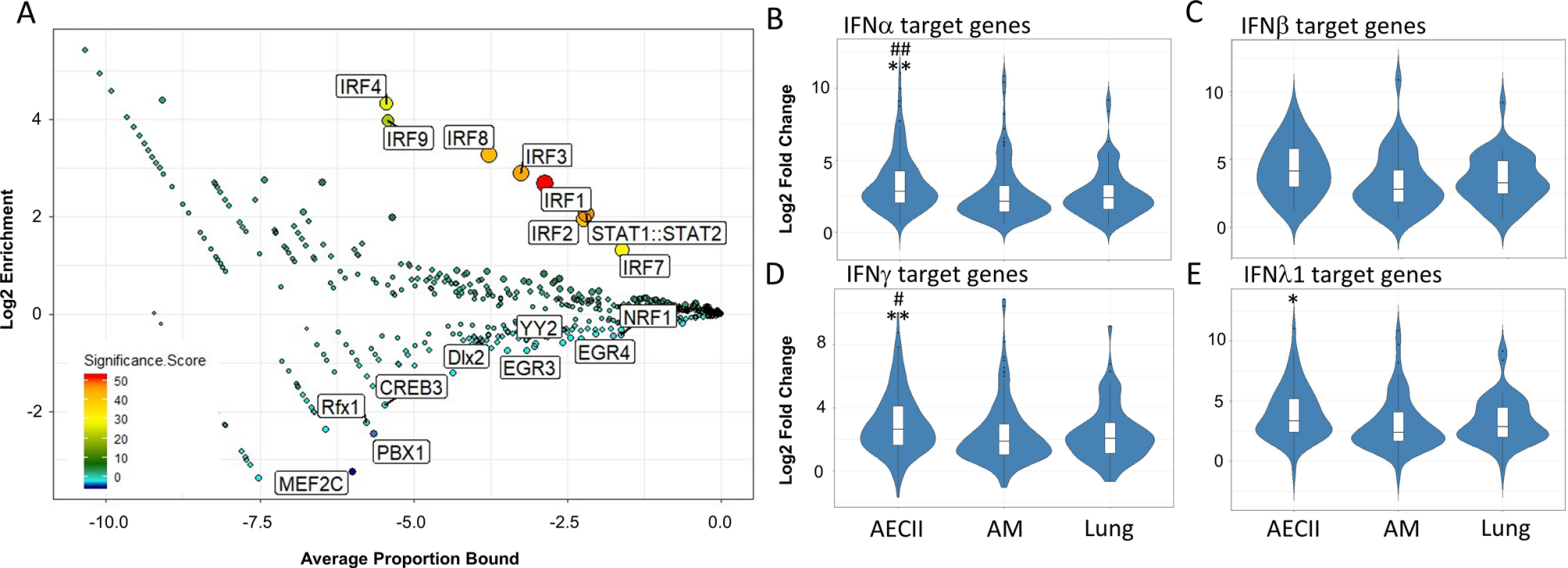
Infection with IAV leads to the broad upregulation of genes in primary human lung cells and in whole lung tissue. (**A**) A set of 302 genes is shared as a common response between all cell types, which is strongly enriched in IRF-responsive genes. Genes that were not regulated in response to IAV served as reference matrix. (**B**–**E**) Sets of IFN target genes as inferred by Ingenuity pathway analysis (IFNγ, n = 139; IFNλ1, n = 64; IFNα, n = 128; IFNβ, n = 41) show a significantly stronger average induction upon viral infection in AECII compared to AM and lung as determined by ANOVA with tukey correction for multiple testing. *p < 0.05, **p < 0.01 versus AM #*p* < 0.05, ##*p* < 0.01 vs. human lung.

Next we used CiiiDER to identify overrepresented and underrepresented transcription factors that may cause the observed gene expression profiles^22^. We probed all cell-specific and overlapping Venn diagram sectors from Fig. 3A. The transcription factors representing the shared fraction of 302 genes were highly enriched for IRFs (IFN response factors, Fig. 4A). For each cell type, there was a unique landscape of transcription factor enrichment and depletion. The AECII-specific gene set yielded a highly significant positive enrichment of the STAT1:STAT2 heterodimer and of IRF2, IRF7 and IRF8. The AM-specific gene set yielded a weak positive enrichment of transcription factors such as Hoxd8 and NFIA. In the human lung, the most enriched transcription factors were ATF1 and E2F4 (Fig. S6).

To identify hallmark events of viral gene induction that shape the observed transcriptional patterns, we extracted gene sets that were upregulated exclusively in one cell type. We grouped the differentially expressed genes by potential upstream regulators as inferred by Qiagen Ingenuity Pathway Analysis. The upstream regulators predicted with the highest confidence were IFNγ and IFNλ (*p* < 1 × 10^–60^ for each overlap). The IFNγ response, illustrated by these target genes (Table S1)**,** was pronounced in AECIIs but significantly weaker in human lung samples and also AMs (Fig. 4D). We compared these data to IFN expression levels in AECIIs and AMs, revealing that AECIIs induce a broader panel of type I IFNs than AMs upon influenza infection (Fig. S7A). We additionally performed ELISA for four prototypic interferons (Figure S7B). The different biological systems secrete different amounts of interferons upon infection, and AECII are the most potent producers of IFNγ RNA and protein. Notably, AECIIs induce IFNλ1 and IFNα, but not IFNβ, target genes to a higher level compared to AM and lung. (Fig. 4B,C,E).

### Correlation with viral transcripts identifies a core set of cellular defense genes

**Figure 5:**
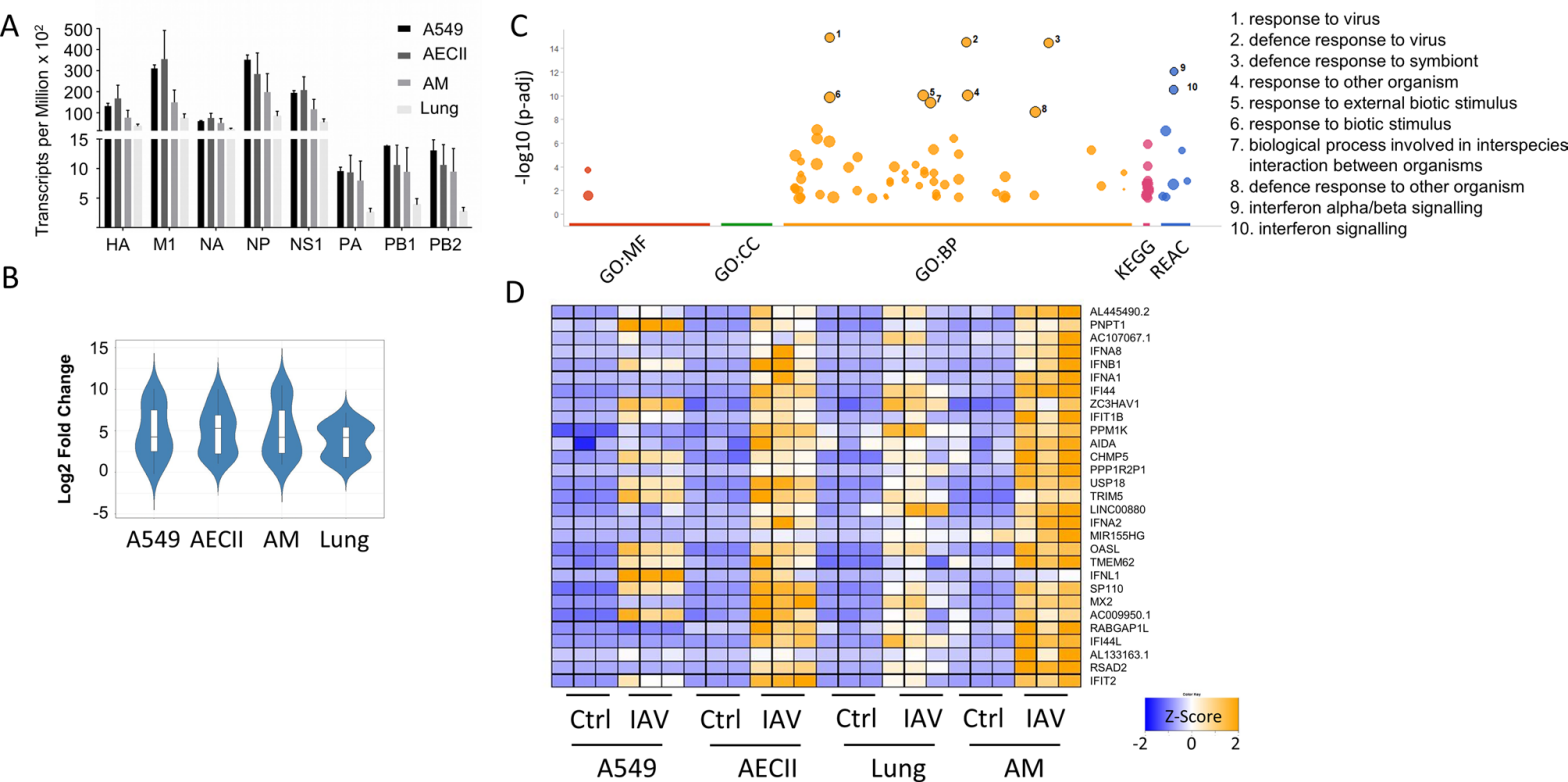
Viral sequences retrieved from infected samples show comparable infection rates across all cell types. Sequences obtained from RNA sequencing were mapped against a hybrid reference genome of human and H3N2 Panama virus. (**A**) Transcripts per million (TPM) and cell type are shown as mean + SEM. Uninfected cells (not shown) showed TPMs below 10 for viral transcripts. (**B**) Genes from the shared upregulated pool (302 genes) that correlate (Pearson > 0.7) with the ratio of viral *NA* mRNA to cellular β-actin (*ACTB*) mRNA (n = 29) show a mean similar induction across all cell types (**B**). These genes show a high enrichment of the cellular defence response pathways (**C**). While the relative induction is similar, abundance across all cell types is different as shown by z-score (**D**). Classifications: BP = biological process, CC = cell compartment, MP = molecular process, KEGG = Kyoto Encyclopedia of Genes and Genomes, REAC = reactome database.

**Figure 6:**
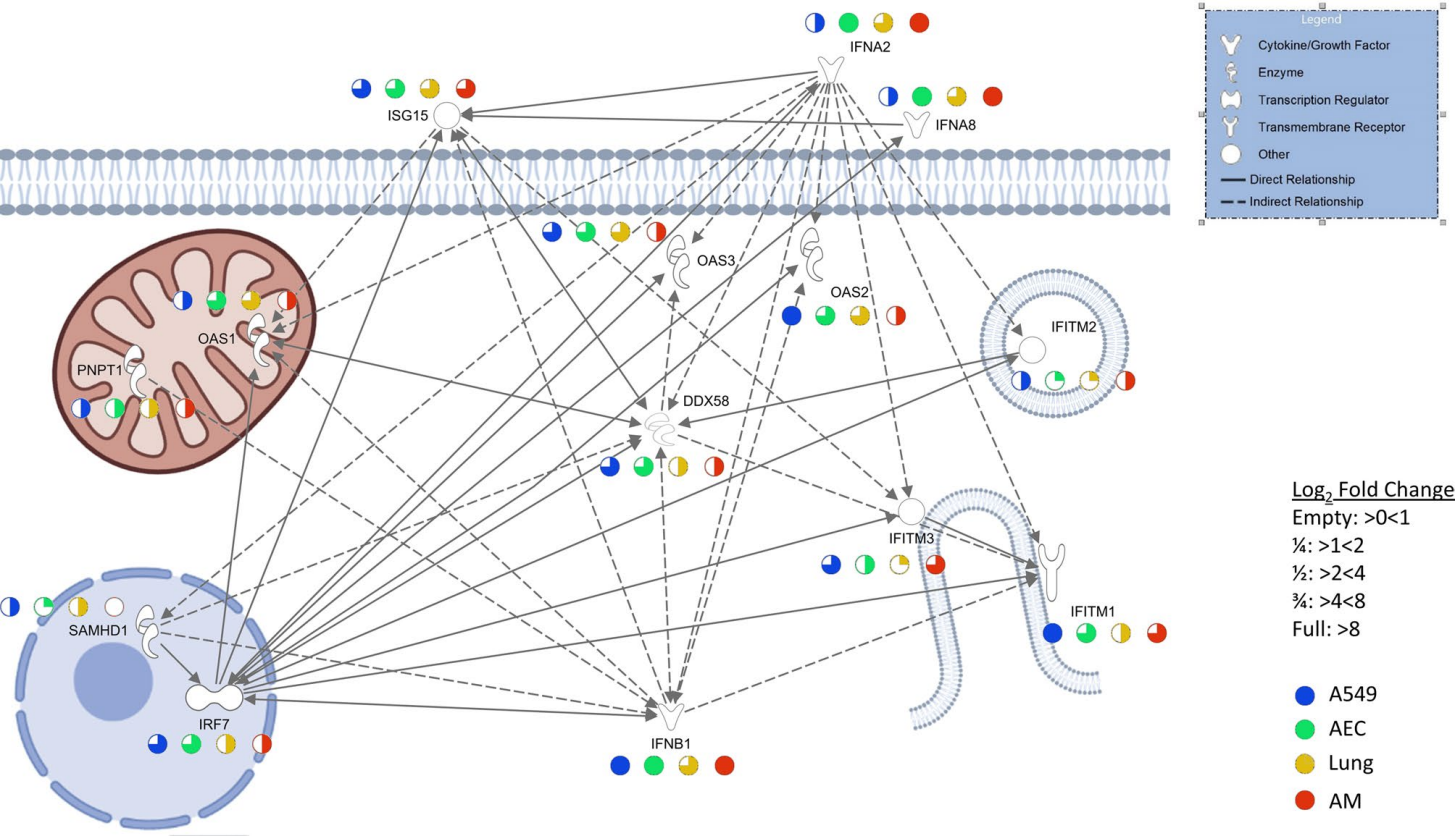
Hallmark genes of the antiviral response are shown in their cellular context with their fold-change regulation in the different biological systems. Details on the interaction between nodes are derived from Qiagen Ingenuity Pathway Analysis and are given in Table S1. Parts of this Figure were made with Biorender.

In order to account for the differences in viral load (Fig. S1) when exploring gene expression, we identified viral transcripts in the infected cells as indicator of viral burden. The abundance (transcripts per million, TPM) of the eight IAV transcripts *HA*, *M1*, *NA*, *NP*, *NS1*, *PA*, *PB1* and *PB2* was broadly comparable across all four sample types, with a tendency towards lower abundance in the lung samples (Fig. 5A). As *NA* was the most evenly expressed viral transcript across all sample types, we normalized the *NA* transcript counts to human *ACTB* transcript (encoding the housekeeping factor β-actin) in order to derive a coefficient that reflects the viral load per sample. We then correlated host gene expression with this ratio. From the pool of shared upregulated genes (Fig. 3A), we extracted 29 candidates that correlated (Pearson Coefficient > 0.7) with this ratio coefficient and showed similar relative induction across cell types (Fig. 5B). For these genes, we observed strong pathway enrichment for cellular defense signaling (Fig. 5C, S8). Looking at these genes individually revealed that, while their mean relative fold change was similar, abundance patterns were different across cell types (Fig. 5D). We summarize expression patterns, interactions and cellular context of selected genes identified in this study in a graphic representation (Fig. 6 and Table S1).

### Correlation of selected genes with published single cell sequencing data

In order to test the fidelity of selected findings from our approach in a single cell sequencing setup, we used a published single cell sequencing dataset that included infection of human lung samples with Pan/99[H3N2]^19^. We can show that correlation of genes that we show to differ between AM and AECII (Fig. 2C) also show poor correlation in the single cell sequencing data (R = 0.28, Figure S9A), while genes that we show to be similar between AM and AECII (Fig. 5D) show a good correlation in the single cell sequencing data (R = 0.83, Figure S9B).

## Discussion

To gain insight into the host response to IAV infection, and specifically the influence of different human cell types and the crosstalk between them^24^, we established a comprehensive map of differential gene expression in primary human AECIIs, AMs, and lung tissue explants under resting conditions and during IAV infection with the Pan/99[H3N2] virus strain. We chose this strain because at the time of experimentation, Pan/99 had been selected by the WHO as a prototypic H3N2 vaccine strain, thereby representing many other circulating IAV strains. Furthermore, there is a reverse genetic system available that will allow to assess the contributions of distinct genetic determinants to the phenotypes of Pan/99 in the infection models of future studies^25^. The sequencing data we obtained from our infection experiments are deposited at NCBI GEO under the accession number GSE206606. Together, our biological models provide insight into the interplay between AMs and AECIIs by allowing the comparison of lung tissue to each cell type cultured in isolation. We also compared primary human AECIIs to the A549 cell line, which is derived from a human alveolar epithelial cell adenocarcinoma and used in many IAV studies. While many of the aspects of cellular responses towards IAV have been studied in the literature^12–14^, this is the first systematic study comparing four different relevant human respiratory host systems by RNA sequencing. This allowed us to establish with high confidence the significant differences in gene expression between A549 cells and AECIIs, as well as broad similarities but also nuanced differences between AECIIs, AMs and human lung samples upon IAV infection. We furthermore measured the viral load in each cell type by quantifying viral transcripts and derived from this a cell-specific transcriptional response to the viral burden, which enabled us to explore key gene expression while accounting for different viral load per sample.

AECIIs are the major target of IAV in the human lung^4,5^. Infection generally results in productive virus replication, whereas immune cell populations (including AMs) tend to be less permissive or even non-permissive to productive replication by seasonal IAV^6^. Furthermore, AECIIs and AMs sense and respond differently to IAV infections. They differ in the sialic acid linkages displayed on the cell surface, which affects their susceptibility to infection^26^, and they produce distinct patterns of inflammatory mediators in response to IAV^27^. The analysis of gene regulation in these cells revealed that the less pronounced relative induction of gene expression in infected AMs reflected the higher basal level of immunoactive genes, e.g. *SAMHD1, ELF1* or *ERAP1*^28–30^. However, the first line of defense against viruses is the production of IFNs^31^, which exert their antiviral functions mainly via the JAK/STAT pathway^32,33^. Interestingly, transcription factor enrichment analysis suggested that STAT1:STAT2 heterodimers were primarily active in AECIIs, correlating with our observation of strong IFN expression in these cells. We may therefore have identified a component of IFN signaling that is mainly active in AECIIs and to a lesser extent in AMs, and which may be an innate feature of the cellular response and/or an indicator that the virus interferes with this response to a different extent in the two cell types.

Primary human AECIIs are known to lose their phenotype and capacity for surfactant synthesis during standard in vitro cultivation^34^. The human pulmonary adenocarcinoma cell line A549 is therefore used extensively as a model, even though its suitability is a matter of discussion^35,36^. We correlated our A549 gene expression data with the public dataset GSE169309, which included a 24 h infection setting of A549 cells with H3N2 influenza. We observed an overall correlation coefficient of R = 0.79 (data not shown), which corroborates the response pattern of our A549 cells to infection. Our further comparison of A549 cells with our primary AECIIs revealed a broadly similar antiviral response in both cell types, but multiple pathways induced in AECIIs showed limited or zero activity in A549 cells because the corresponding genes were not induced by infection. As examples, we observed the non-expression of *IFNA2* and *IFNA8* (*antiviral mechanism by IFN-stimulated genes*) and *IFITM1* and *IFITM2* (*RIG-I like receptor signaling*). This indicates that A549 cells mount a more limited cytokine response than primary cells, and have a diminished capacity to sense the virus via RIG-I and respond with an appropriate IFN signal. On the other hand, A549 cells show comparable or even increased gene induction in comparison to AECII, *e.g.* in the case of *DDX58* (*RIG-I*), *ISG15, IRF7*, and *PNPT1*, indicating their partially intact response pattern to IAV infection.

A clear limitation of our study is the bulk nature of our sequencing data. While we believe that the bulk approach has its advantages over single cell sequencing, notably sparing the cells from the physical stress of separation and less room for data misinterpretation due to lower complexity, we clearly do not achieve the level of resolution that single cell sequencing would have provided. Therefore, we show the induction of two sets of key genes identified in our study in a single cell sequencing dataset. A further intriguing aspect that goes beyond the scope of this study is the dependency of the virus life cycle and gene expression regulation on the chosen biological host system. As our paper highlights the cell-type specific differences in the host response, we expect that these differences also impact on viral gene expression and viral assembly. Furthermore, to maintain consistency and reproducibility, and to interpret findings in the proper context, it is essential to take into account the limitations of 2D cell cultures when using them to model aspects of organ function. Besides AECII and AM, ex vivo cultured alveolar tissue contains at least AECI and microvascular endothelial cells as well as resident immune cells, which may impact on the IAV-related tissue activation in situ. Therefore, we also investigated the gene expression patterns in whole lung human tissue. We established a core signature that was shared by all primary models, and on the basis of these transcripts compared all three models by PCA. PC1 separated uninfected from infected samples, PC2 separated human lung tissue from AECII, and PC3 separated AECII from AM. In order to account for the markedly reduced replication of virus in the human lung, we focused on the genes driving PCA separation and found *IFITM1, IFITM2* and *IFITM3* to be particularly abundant in the human lung. As this gene family has reported antiviral properties^37^, they might explain partly why the herein used IAV strain showed lesser growth in whole lung tissue. RelA, which has the highest induction and abundance also in the lung samples might also account for this observation, as it enables NF-κB activation and subsequent pro-inflammatory gene transcription^38^. Furthermore, ARID5A, which we highlight as most abundant in the human lung, has been described to stabilize transcripts such as *IL6*, *STAT3* and *TNFRSF4*^39^, which we have found in the highest abundance in the human lung, albeit not further induced by IAV infection. On the other hand, the transcripts of *OAS1*, *OAS2* and *OAS3*, which are also described as antiviral^40^, show a lesser abundance in the human lung compared to AECII. Likewise, the lung showed the lowest abundance and induction of *IFNB1* upon IAV infection. These findings illustrate the differential transcription status of core signature genes in the different primary models. We furthermore found the abundant transcript for the dendritic cell maturation factor FLT3LG which suggests the presence of dendritic cells^41^ that contribute to the distinct transcriptional profile of the complete tissue compared to isolated cell types.

Viral transcripts were detected as indicator of the viral burden in the infected cells and their abundance was correlated with host gene expression. We observed a comparable relative induction of host genes that correlated with viral burden across all cell types, but we also saw less abundance of these genes in human lung tissue and A549 cells. This again argues for the complex crosstalk in the human lung tissue which might exert inhibitory effects on gene expression, and it highlights the shortcomings of isolated cells. Usage of 2D cell culture of isolated primary cells and in particular cell lines comes with severe limitations because of the lack of 3D tissue integrity. Intact tissue and in the future, organoids, represent a much more physiological model to study ongoing infection^42–44^. We have comprehensively mapped the shared and exclusive transcriptional responses to IAV H3N2 infection in AMs, AECIIs, A549 cells, and importantly, human lung tissue. The similarities and differences we report will assist careful selection of cell culture models and their performance.

## Supplementary Information

Supplementary Figure S1.

Supplementary Figure S2.

Supplementary Figure S3.

Supplementary Figure S4.

Supplementary Figure S5.

Supplementary Figure S6.

Supplementary Figure S7.

Supplementary Figure S8.

Supplementary Figure S9.

Supplementary Table S1.

## Data Availability

RNA sequencing data are deposited at NCBI GEO under the accession number GSE206606 (https://www.ncbi.nlm.nih.gov/geo/query/acc.cgi?acc=GSE206606).
