# Supplementary figures and images for: Transcriptomic comparison of primary human lung cells with lung tissue samples and the human A549 lung cell line highlights cell type specific responses during infections with influenza A virus
Source: Sci Rep. 2022 Nov 29;12:20608. doi: 10.1038/s41598-022-24792-4 (PMC9709075; doi:10.1038/s41598-022-24792-4)

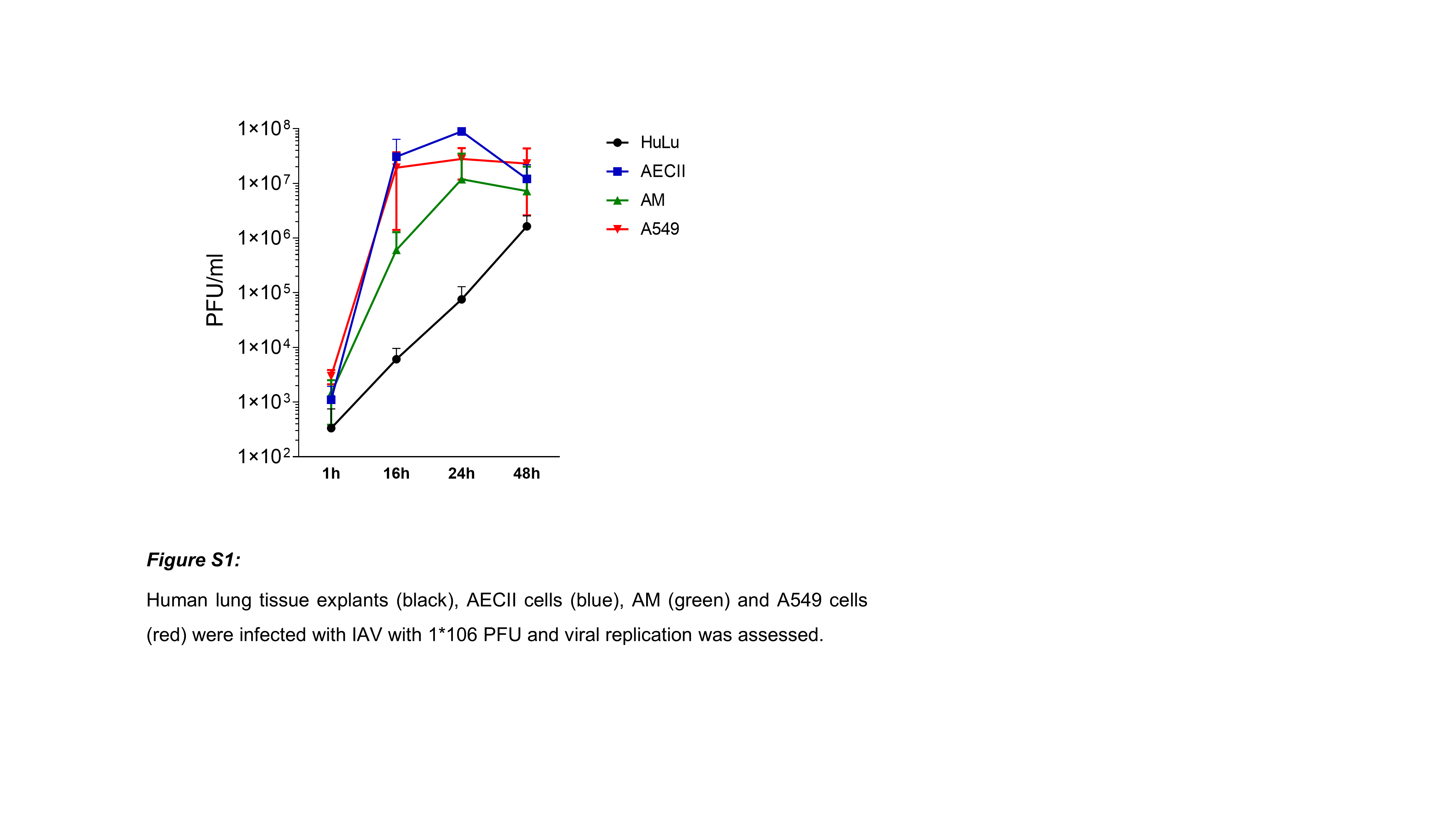

Supplement: Supplementary file 1 — Supplementary Figure S1. [file 41598_2022_24792_MOESM1_ESM.tif]

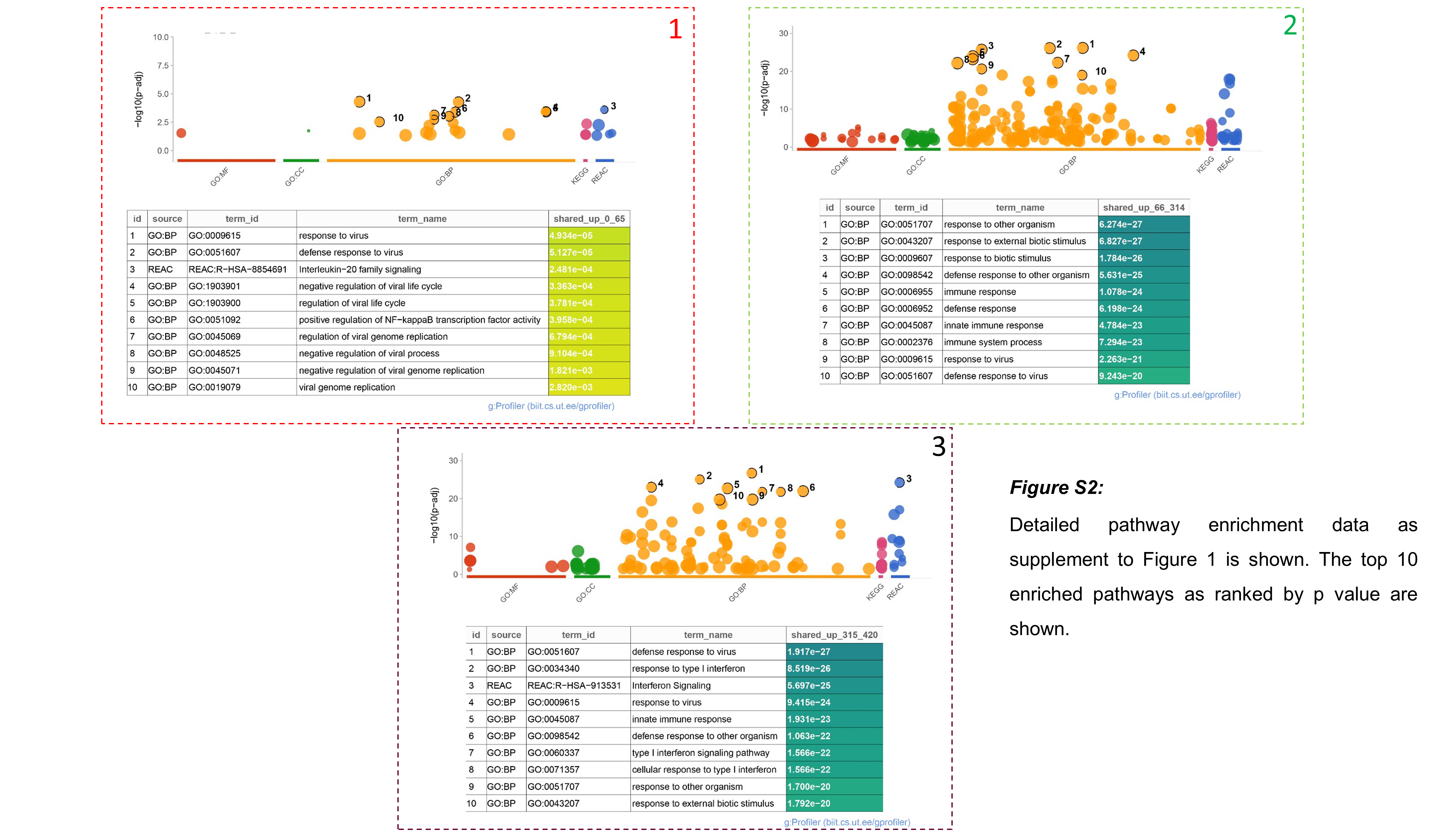

Supplement: Supplementary file 2 — Supplementary Figure S2. [file 41598_2022_24792_MOESM2_ESM.tif]

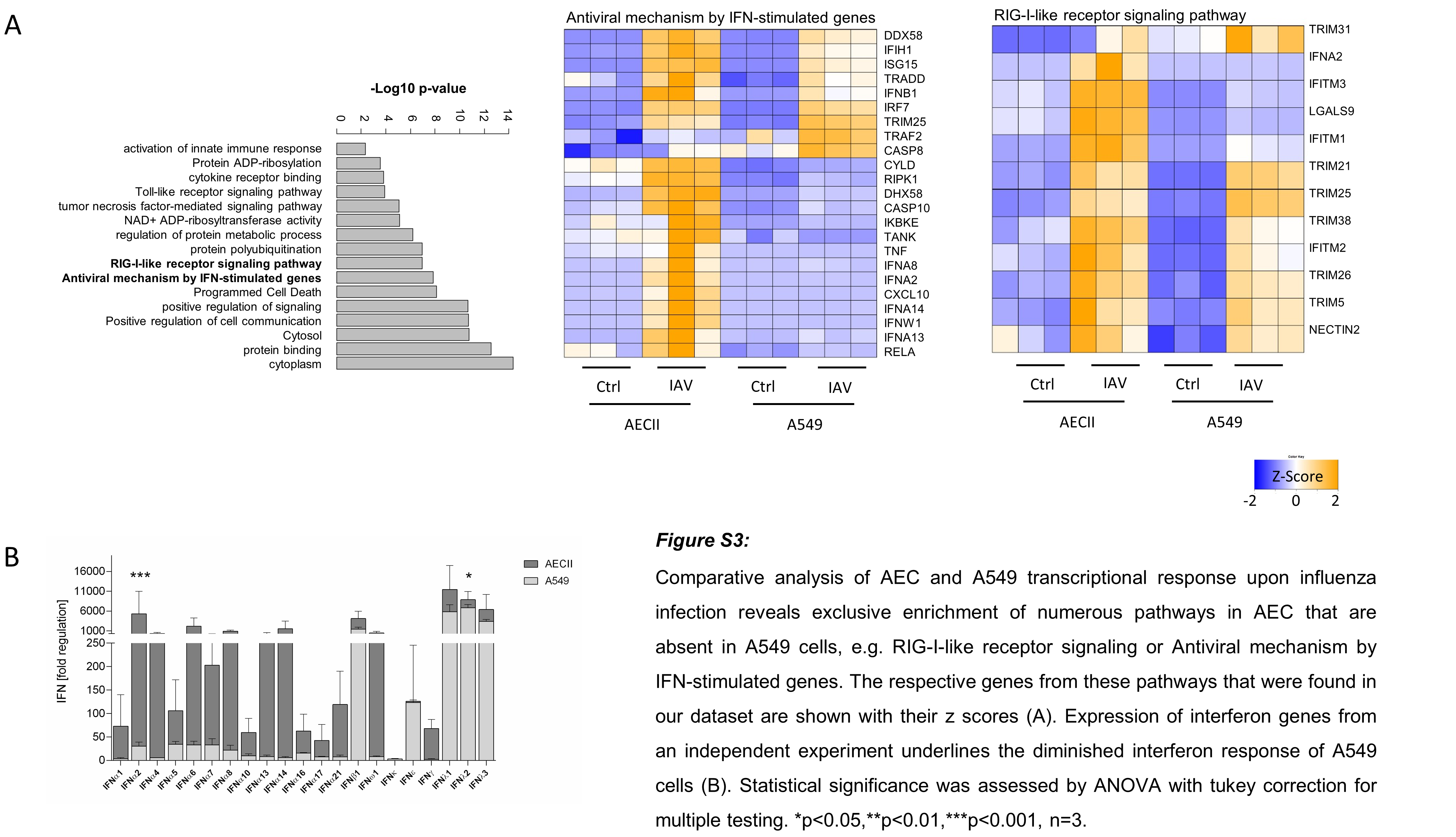

Supplement: Supplementary file 3 — Supplementary Figure S3. [file 41598_2022_24792_MOESM3_ESM.tif]

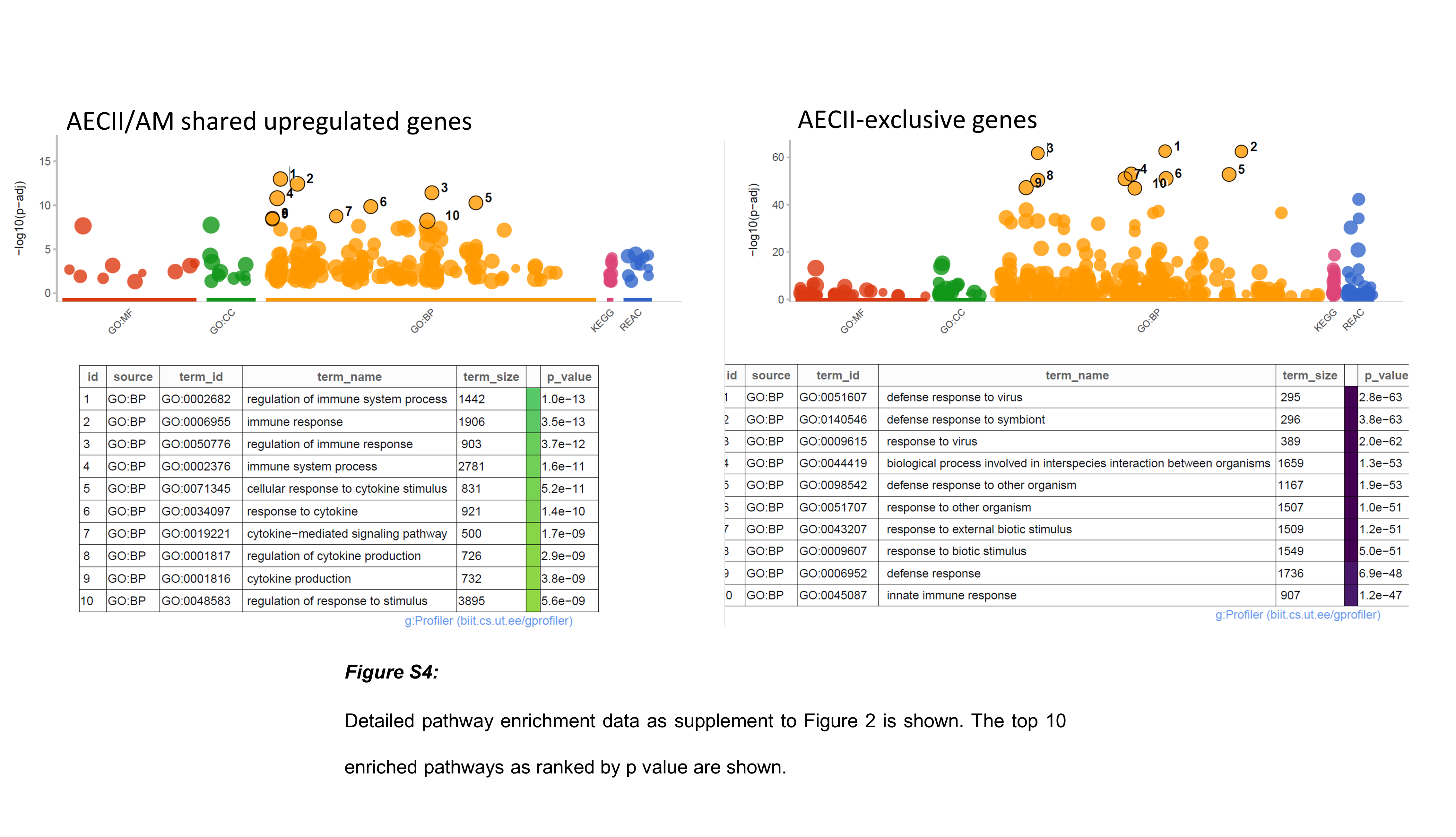

Supplement: Supplementary file 4 — Supplementary Figure S4. [file 41598_2022_24792_MOESM4_ESM.tif]

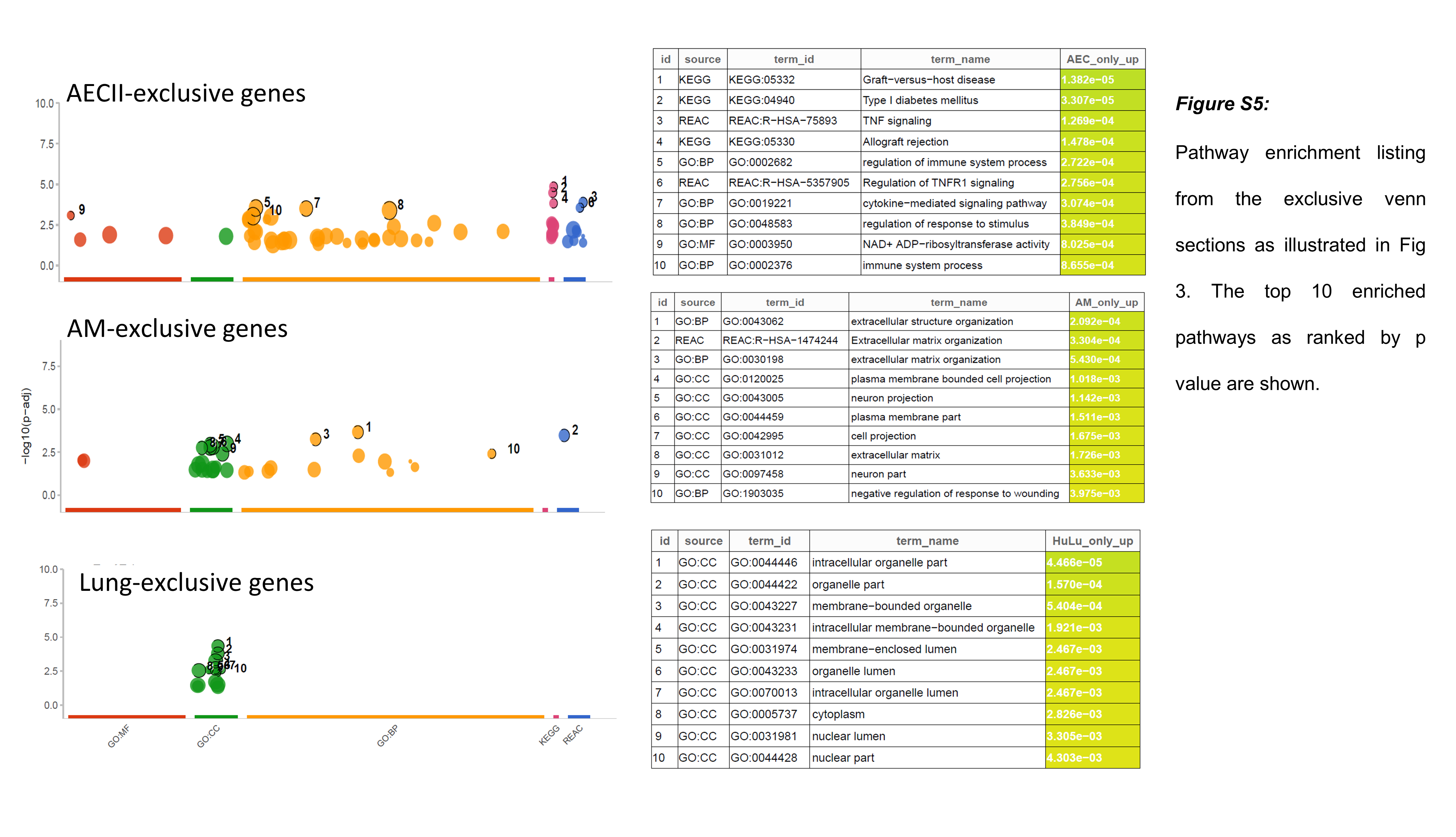

Supplement: Supplementary file 5 — Supplementary Figure S5. [file 41598_2022_24792_MOESM5_ESM.tif]

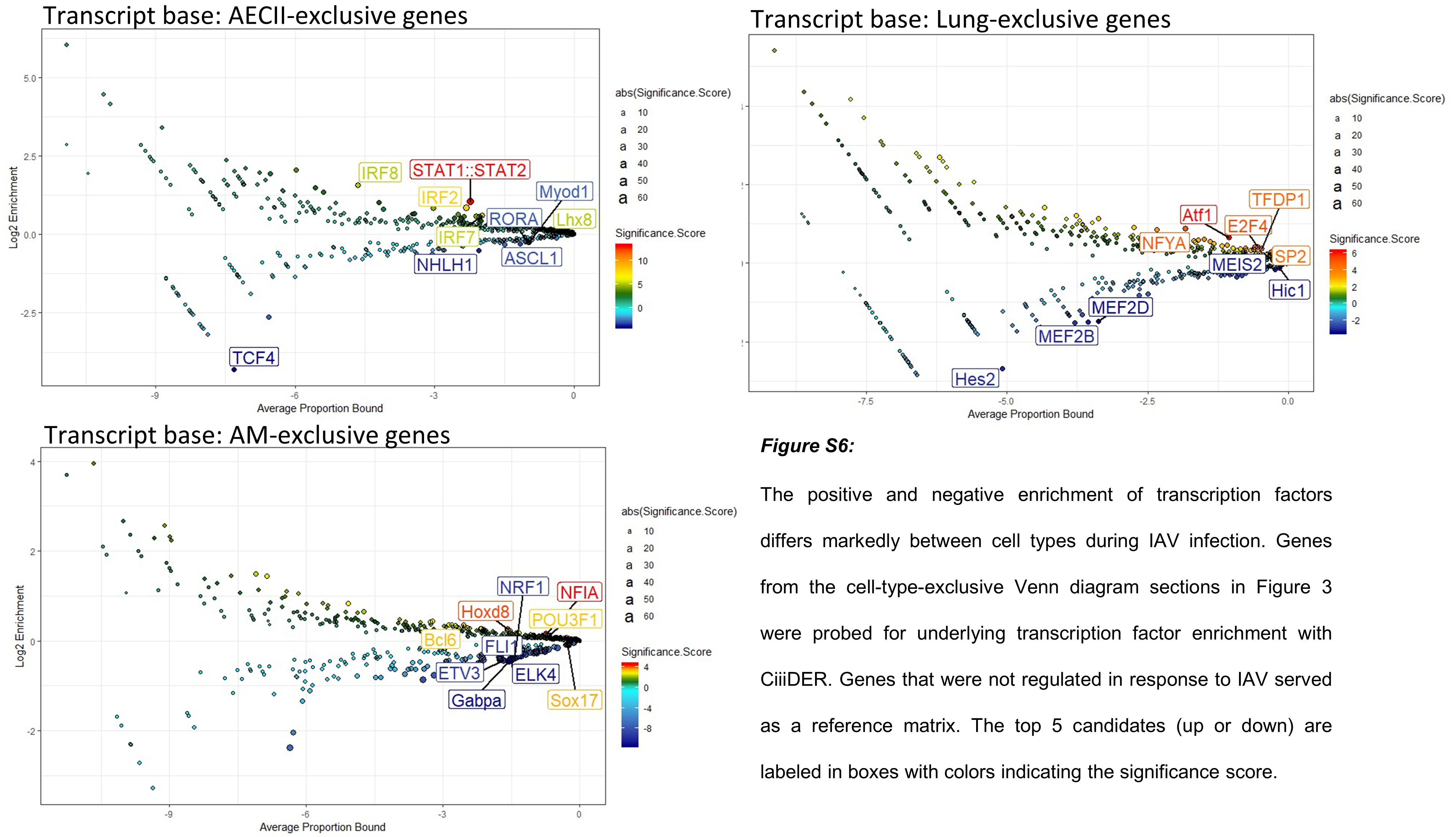

Supplement: Supplementary file 6 — Supplementary Figure S6. [file 41598_2022_24792_MOESM6_ESM.tif]

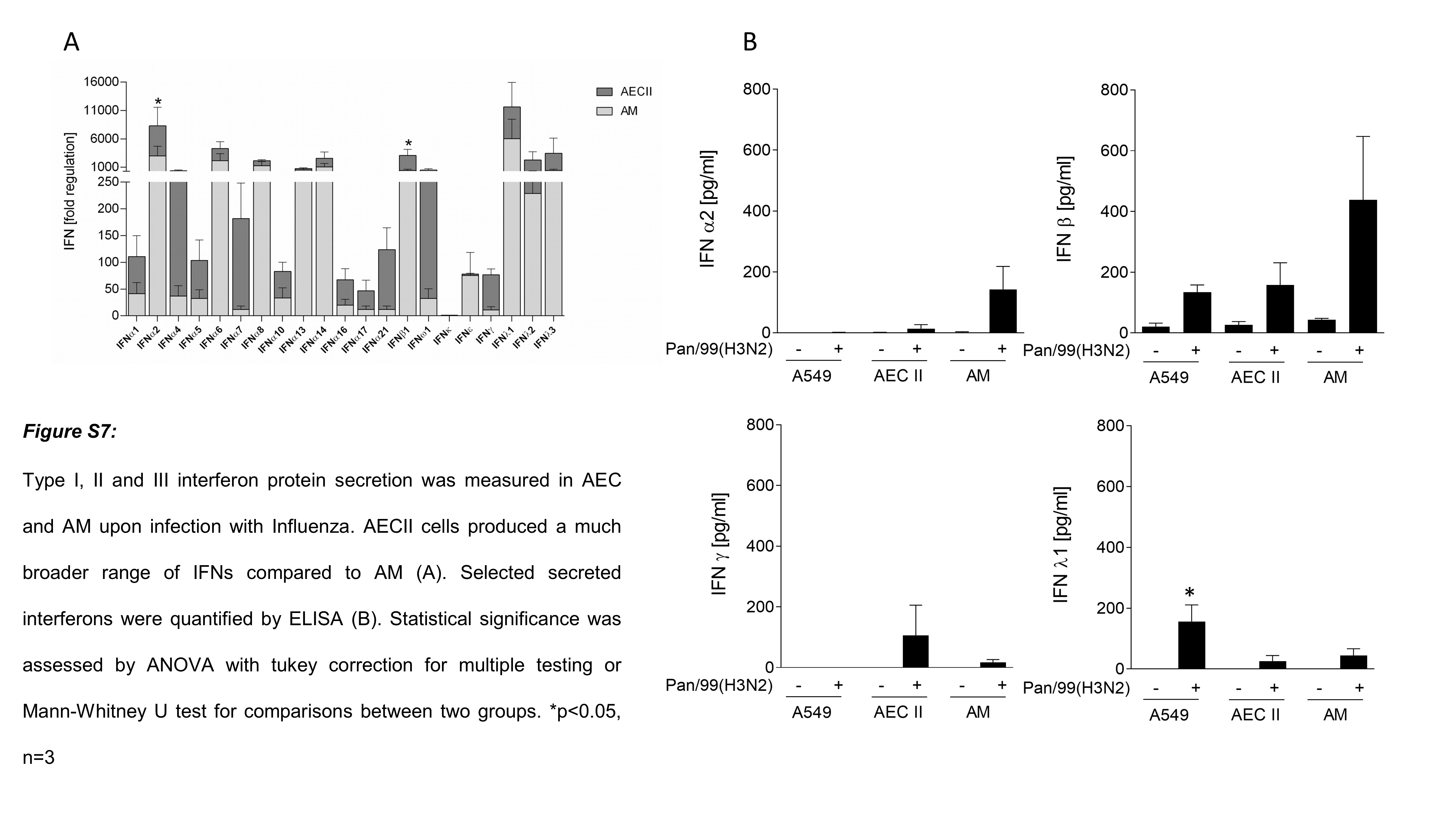

Supplement: Supplementary file 7 — Supplementary Figure S7. [file 41598_2022_24792_MOESM7_ESM.tif]

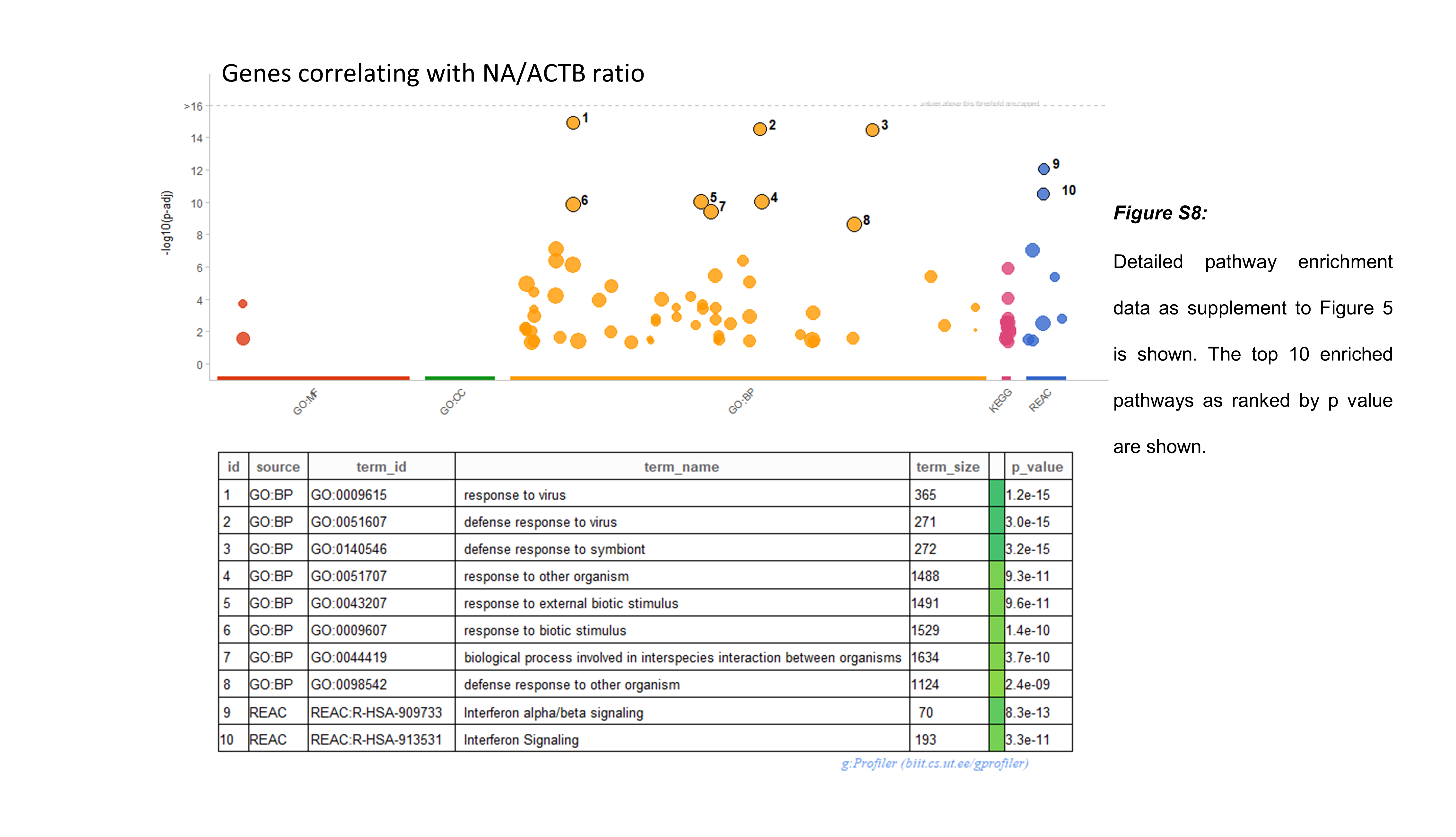

Supplement: Supplementary file 8 — Supplementary Figure S8. [file 41598_2022_24792_MOESM8_ESM.tif]

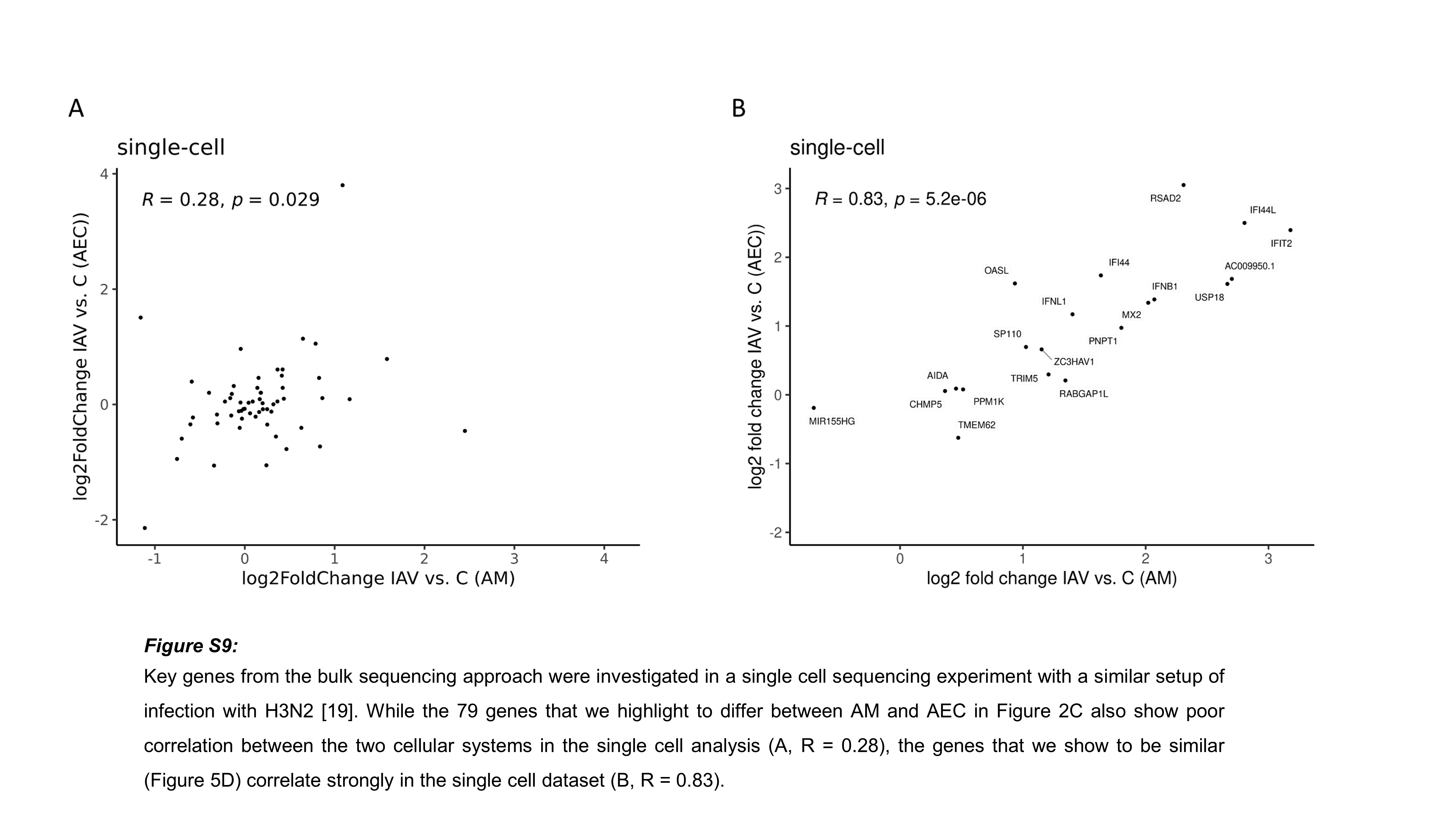

Supplement: Supplementary file 9 — Supplementary Figure S9. [file 41598_2022_24792_MOESM9_ESM.tif]
